# Breast cancer in Ethiopia: evidence for geographic difference in the distribution of molecular subtypes in Africa

**DOI:** 10.1186/s12905-018-0531-2

**Published:** 2018-02-14

**Authors:** Endale Hadgu, Daniel Seifu, Wondemagegnhu Tigneh, Yonas Bokretsion, Abebe Bekele, Markos Abebe, Thomas Sollie, Sofia D. Merajver, Christina Karlsson, Mats G. Karlsson

**Affiliations:** 1 Department of Biochemistry, School of Medicine, Addis Ababa University, Addis Ababa, Ethiopia; 2 Department of Oncology, School of Medicine, Addis Ababa University, Addis Ababa, Ethiopia; 3 Department of Pathology, School of Medicine, Addis Ababa University, Addis Ababa, Ethiopia; 4 Department of Surgery, School of Medicine, Addis Ababa University, Addis Ababa, Ethiopia; 5 Armauer Hansen research Institute (AHRI), Addis Ababa, Ethiopia; 6 Dept of Laboratory Medicine, Faculty of Medicine and Health, Orebro University, Orebro, Sweden; 7 School of Health Sciences, Orebro University, Orebro, Sweden; 8 University of Michigan Comprehensive Cancer Center, Ann Arbor, MI USA

**Keywords:** Breast cancer, Molecular subtypes, Ethiopia, Africa

## Abstract

**Background:**

Breast cancer is a heterogeneous disease with several morphological and molecular subtypes. Widely accepted molecular classification system uses assessment of estrogen receptor (ER), progesterone receptor (PR), human epidermal growth factor receptor 2 (HER2) and proliferation marker Ki67. Few studies have been conducted on the incidence and molecular types of breast cancer in Sub-Saharan Africa. Previous studies mainly from Western and Central Africa, showed breast cancer to occur at younger ages and to present with aggressive features, such as high-grade, advanced stage and triple-negative phenotype (negative for ER, PR and HER2). Limited data from East Africa including Ethiopia however shows hormone receptor negative tumors to account for a lower proportion of all breast cancers than has been reported from elsewhere in Africa.

**Methods:**

In this study from Tikur Anbessa Specialized Hospital, 114 breast cancer patients diagnosed between 2012 and 2015 were enrolled. ER, PR, Ki67 and HER2 receptor status were assessed using immunohistochemistry from tissue microarrays. FISH was used for assessment of gene amplification in all equivocal tumor samples and for confirmation in HER2-enriched cases.

**Results:**

The distribution of molecular subtypes was: Luminal A: 40%; Luminal B: 26%; HER2-enriched: 10%; TNBC: 23%. ER were positive in 65% of all tumors and 43% the cases were positive for PR. There was statistically significant difference in median age at diagnosis between the molecular subtypes (*P* < 0.05). There was a bimodal distribution of molecular subtypes in different age ranges with Luminal B subtype being more common at younger ages (median = 36) and Luminal A subtype more prevalent at older ages (median = 42). There were no statistically significant differences in tumor grade, histology, and stage between the molecular subtypes of breast cancer.

**Conclusion:**

The present study detected Luminal A breast cancer to be the most common subtype and reveals a relatively low rate of hormone receptor negative and TNBC. Our findings and results from other East African studies suggest geographic variability in the distribution of the molecular subtypes of breast cancer in Africa and hence have important clinical and policy implications for breast cancer control and treatment in Ethiopia.

## Background

Breast cancer is the most common malignant neoplasm among women in Addis Ababa (Ethiopia). The Addis Ababa Cancer registry reports that breast cancer accounts for 34% of all female cancer cases, followed by cervical cancer at 16% [1]. Although breast cancer has a markedly higher incidence in developed countries, half of new breast cancer diagnosis and about 60% of breast cancer deaths occur in the developing world [2].

A significant increase in breast cancer incidence is reported in most Sub-Saharan Africa countries suggesting an increasing public health problem in a continent with existing infrastructures having been developed mainly for maternal, child health and infectious diseases [3]. Widespread urbanization, changing patterns of reproductive and environmental risks factors, obesity, decreased physical activity, and increasing life expectancy [3, 4] are among the salient factors implicated in the steady rise in breast cancer incidence across low income countries.

Breast cancer is a heterogeneous disease with different morphological and molecular subtypes [5, 6]. The morphological classification is still the foundation of histopathological diagnosis, but in the era of modern personalized medicine, a number of molecular classification systems have been introduced. In this context, estrogen receptor (ER) and progesterone receptor (PR) analysis in breast carcinomas were the first established biomarker assays with both prognostic and predictive power and they have been in use since the 1970s [7]. Human Epidermal Growth Factor 2 (HER2) was discovered in the 1990s and HER2-targeted therapy was subsequently introduced; the introduction of targeted antibodies that interfere with HER2 signaling followed and their use has led to improved survival among breast cancer patients whose tumors overexpress HER2 [8].

Further studies have revealed distinct molecular subtypes of breast cancer based upon the gene expression profiling of tumors [9]. Since classifications based on gene expression are still considered unaffordable in most global settings outside high income countries of the Western hemisphere, immunohistochemistry (IHC) studies have gained widespread acceptance as potential robust surrogates for more complex bar-code type assays. A combination of ER, PR, and HER2 defines the molecular classes initially identified by gene expression profile [10]. The classification systems used by Nielsen [11], Sotiriou [12] and the St. Gallen international panel of experts [13] in 2004, 2006, and 2011 respectively are widely used among research communities. The St. Gallen international panel of experts incorporated the well-established proliferation-associated antigen Ki67 that may also provide additional prognostic and predictive value to the previous classification systems [13, 14]. Several studies show that molecular subtypes of breast cancer vary substantially in their behavior and response to treatment [15]; however, at present none of these schema are widely used outside of Western countries.

With increasing opportunities for implementing modern health care in Sub-Saharan African countries, more in-depth knowledge of African breast cancer molecular characteristics compared to the more studied western countries is essential for improving prognosis and management of breast cancer.

Few studies have been conducted in Africa on the clinicopathological and biological characteristics of breast cancer in Sub-Saharan African countries. The published studies conducted so far exhibit some degree of divergence in their results. The majority of these studies, reviewed by Brinton et al., were mainly carried out on west and central Africa and reported that breast cancer patients exhibit specific features compared to the western countries [16]. According to these authors, breast cancer in Sub-Saharan Africa is reported to occur in younger age and show more aggressive features such as high-grade tumors and triple-negative phenotypes (negative for expression of ER and PR and for over-expression of HER2) [16]. Similar patterns to these African studies have been reported among African American women with disproportionately high incidence of triple negative phenotype as compared to White Americans [17–19]. Preliminary evidence, however, shows geographical diversity in the distribution of breast cancer molecular subtypes in Sub-Saharan African region with the rate of triple negative breast cancer reported to be relatively lower in East Africa [20–22]. In the present study, we aimed to study a large collection of cases from a major Ethiopian institution where women are treated for breast cancer and investigate the molecular subtypes using the most recent receptor-based classification [13, 14].

## Methods

### Case selection

We performed a cross sectional, retrospective study of women diagnosed with breast cancer that were treated at the Oncology Centre in Tikur Anbessa Specialized Hospital (TASH). The TASH is a reference centre for cancer treatment in Ethiopia located in the capital Addis Ababa, and it receives patients from all regions in the country. The patients enrolled in the study consisted of women with available archived surgical specimen at TASH or St. Paul’s Hospital Millenium Medical College (SPHMMC) which is also a referral hospital in the capital providing surgical service to breast cancer patients in the country. Information about demographic data and tumor characteristics were obtained from the patient medical records at TASH. The collected variables were age, tumor type, grade and stage of disease.

Only patients having undergone surgery between 2012 and 2015 were included in the study because specimens collected from patients before this period were assessed to be missing or improperly stored at the hospitals. One hundred eighty-nine patients were initially included in the study but 66 cases were excluded because formalin-fixed paraffin-embedded (FFPE) tissue was not available in the pathology departments or the biopsy was not of sufficient quality for the study.

The final cohort in the study consisted of 123 breast cancer patients. All archived FFPE blocks were sectioned and H&E stained and examined by a pathologist (TS) for locating tumors in the block to be used for constructing tissue microarray (TMA). After TMA construction 114 cases were judged to be evaluable.

### Tissue microarray

Digital images for constructing tissue microarray (TMA) were taken from slides scanned with a Pannoramic 250 digital scanner (3D HISTECH Ltd., Budapest, Hungary) and representative areas selected from images using the software program ‘Case viewer’ (3D HISTECH Ltd., Budapest, Hungary). TMA were constructed using the TMA grand master automated system (3DHISTECH Ltd., Budapest, Hungary). 0.6 mm punch biopsies in triplicate corresponding to the marked area were taken from donor paraffin blocks and merged into TMA recipient paraffin blocks.

### Immunohistochemistry

Immunohistochemistry (IHC) was done in automated system using the Dako Autostainer Link. Formalin fixed, paraffin sections were cut at 4 μm and rehydrated to water. Heat induced epitope retrieval was performed with FLEX TRS High pH Retrieval buffer for 20 min. After peroxidase blocking, the specific monoclonal antibodies were applied at room temperature for 20 min. The FLEX + Rabbit EnVision System was used for detection. DAB chromogen was then applied for 10 min. Slides were counterstained with Mayers hematoxylin for 5 s and then dehydrated and coverslipped. Slides were scanned on a Pannoramic 250 digital scanner (3D HISTECH Ltd., Budapest, Hungary) and images scored using the software program ‘Case viewer’ (3D HISTECH Ltd., Budapest, Hungary). Negative controls were included in each staining run.

### Sources and dilutions of primary antibodies

ER clone EP1, PR clone PgR1294, Ki67 clone Mib-1 and HER2 Herceptest, all from Agilent Dako, USA and all Ready-to-use were used.

### Fluorescence in situ hybridization

Slides were deparaffinized, rinsed in absolute alcohol, and air dried. The sections were then subjected to pretreatment according to the manufacturer’s protocol. Slides were hybridized with a probe mix in HYBrite (Vysis, Des Plaines, IL) where denaturation was set at 6 min at 73 °C and hybridization for 17 h at 37 °C. Probe mixes used were PATHYVISION (HER-2/CEP17) FISH Probe Kit from Abbott Molecular, Des Plaines, IL. Slides were scanned on a Pannoramic MIDI digital scanner (3D HISTECH Ltd., Budapest, Hungary) and images scored using the software program ‘Case viewer’ (3D HISTECH Ltd., Budapest, Hungary). 20 tumor cells were evaluated for scoring.

### Scoring of each marker

Tumors were considered positive for ER and PR when at least 1% of the tumor cells showed nuclear staining irrespective of intensity according to ASCO/CAP 2013 guidelines [23]. HER2 was graded based on recommendations from Fitzgibbons et al. [24]. Grading for HER2 is based on the degree of membrane staining, on a scale of 0–3+. Grades of 0–1+ are considered negative, a grade of 2+ is equivocal, and a grade of 3+ is considered positive for HER2 labeling. Specimens with a HER2:CEP17 ratios of ≥2:0 were scored as positive for HER2 amplification (FISH-positive), and specimens with a HER2:CEP17 ratio of < 2:0 were scored as negative for HER2 amplification (FISH-negative). For Ki67 evaluation a minimum of 500 cells were counted in hot spots. A Ki67 cut-off point of 20% was defined as high according to the St. Gallen international panel of experts’ recommendation [14].

### Molecular sub-typing

Breast carcinoma was classified into the following four sub-types according to St. Gallen international expert’s consensus 2013 [14]: luminal A (ER and/or PR-positive, HER2-negative and Ki67 < 20%), luminal B (ER and/or PR-positive, HER2-positive OR ER and/or PR-positive, HER2-negative and Ki67 ≥ 20%), HER2-enriched (ER and PR-negative, HER2 positive) and triple-negative (ER-negative, PR-negative and HER2-negative).

HER2-enriched cases were confirmed by FISH analysis.

### Statistical analysis

Statistical analysis was done using SPSS for windows version 21.Continous data are reported as mean ± SD or Number (proportions). Skew distributions are reported as the median value with minimum and maximum. All *P* values are two tailed and P value < 0.05 was considered statistically significant. Chi-square test and ANOVA were used to determine the correlations.

## Results

### Patients

There were 114 participants with evaluable samples in this study. Mean age at diagnosis was 43 years (SD 14) and median age was 40 (range 22–75). Most of the participants (40%) were < 40 years old. About 31% of the participants were ≥50 years and only 19% were 40–49 years old. Table 1 shows basic pathological and molecular characteristics of the study subjects. ER positive tumors represent 65% of the cases (Table 1). There was no statistically significant difference in clinicopathological characteristics between ER positive and ER negative tumors (Table 2).

**Table 1:** Baseline clinicopathological and molecular characteristics of the study subjects (*n*=114)

Variables	N(%)
Histological Grade	
I	7(6)
II	32(28)
III	39(34)
Missing	36(32)
Estrogen Receptor	
Positive	74(65)
Negative	40(35)
Progesterone Receptor	
Positive	49(43)
Negative	64(56)
Missing	1(1)
HER2	
Positive	26(23)
Negative	87(76)
Missing	1(1)
Histological Type	
Infiltrating Ductal	67(59)
Lobular	6(5)
Others/Not classified	25(22)
Missing	16(14)
Stage	
I	19(17)
II	37(33)
III	36(31)
IV	4(3)
Missing	18(16)

**Table 2:** Association between ER expression and clinicopathological parameters among the study participants (*n* = 114)

Clinico-pathological parameters	ER Positive	ER Negative	Total	*p*-value
Median age at Diagnosis(min-max)	42(22–75)	41(27–65)		0.284
Tumor Grade, n (%)				
I	5(10)	2(7)	7(9)	
II	18(36)	14(50)	32(41)	0.480
III	27(54)	12(43)	39(50)	
Stage, n (%)				
I	13(20)	6(19)	19(20)	
II	27(42)	10(31)	37(39)	0.639
III	22(34)	14(44)	36(37)	
IV	2(3)	2(6)	4(4)	
Histological Type, n (%)				
Infiltrating ductal	41(60)	26(80)	67(68)	0.123
Lobular	6(10)	0(0)	6(6)	
Others/Not classified	18(30)	7(20)	25(26)	

### Distribution of molecular subtypes

A total of 112 cases had complete data concerning IHC. These cases were classified according to St. Gallen international classification system 2013 [14]. Table 3 shows distribution of the molecular subtypes. The largest proportions of cases were classified as Luminal A (40%). Triple negative breast cancer represents 23% of all cases.

**Table 3:** Molecular Subtypes of breast Cancer

Molecular Subtypes	Cases, n	Percent
Luminal A	45	40
Luminal B	30	26
HER2-enriched	11	10
Triple Negative/Basal like	26	23
Missing	2	2
Total	114	100.0

**Fig. 1:**
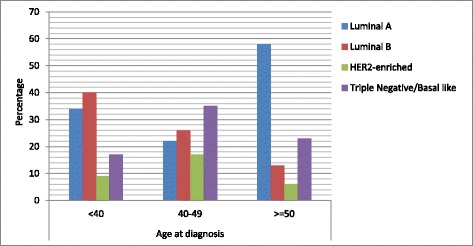
Percentage distribution of the molecular subtypes of breast cancer in different age groups. The figure shows a Luminal B peak for participants in < 40 age range and Luminal A peaks in the age range > =50. Triple negative/basal-like breast cancer is more common among participants between the age of 40 and 49

The median ages at diagnosis across the molecular subtypes were statistically significantly variable (*P* < 0.05) with 42, 36, 40, and 45 for Luminal A, Luminal B, HER2-enriched and TNBC TNBC. Table 4 and Fig. 1 show an earlier onset of luminal B compared to the other molecular subtypes and compares frequencies across the molecular subtypes. The frequencies of the different molecular subtypes were statistically significantly different in different age ranges (*P* < 0.05). It also reveals a decline in luminal B subtypes as patients age increase with the incidence of luminal A exceeding it at the age ranges > = 50.

**Table 4:** Frequency distribution of the molecular subtypesof the study participants in different age range

Age at diagnosis	Molecular Subtypes					p-value
	Luminal A	Luminal B	HER2-enriched	Triple Negative/ basal-like	Total	
< 40	16	19	4	8	47	0.031
40–49	5	6	4	8	23	
> = 50	18	4	2	7	31

Table 5 shows the distribution of clinicopathological parameters in each molecular subtype. There was no statistically significant difference in the distribution of the clinicopathological parameters between the different molecular subtypes of the tumors.

**Table 5:** Distribution of Clinicopathological parameters in each molecular subtypes of breast cancer among the study participants

Clinico-pathological Parameters	Luminal A	Luminal B	HER2-enriched	Triple Negative	Total	p-value
Median age at diagnosis(min-max)	47(22–75)	35(22–53)	41(27–65)	46(29–60)		0.009
Tumor grade, n (%)						
I	5(18)	0(0)	1(13)	1(6)	7(9)	
II	11(39)	8(36)	5(63)	7(39)	31(41)	0.243
III	12(43)	14(64)	2(25)	10(55)	38(50)	
Stage, n (%)						
I	9(24)	5(19)	1(11)	4(18)	19(20)	
II	13(35)	13(48)	1(11)	9(41)	36(38)	0.632
III	14(38)	8(30)	6(67)	8(36)	36(38)	
IV	1(3)	1(4)	1(11)	1(5)	4(4)	
Histological type, n (%)						
Ductal	29(70)	15(60)	7(80)	17(80)	66(69)	0.708
Lobular	2(10)	4(10)	0(0)	0(0)	6(6)	
Others/Unknown	9(20)	8(30)	2(20)	5(20)	24(25)	

## Discussion

Progress in molecular research have led to the classification of breast cancers into distinct subgroups (luminal, normal breast-like, Her2/neu-positive and basal-like subtypes) based on mRNA expression profiles [25]. However, gene expression profiling using cDNA microarray or RNA seq technology is not currently feasible in clinical settings due to its high cost and technical complexity. Therefore, IHC markers have been validated and used as surrogates for cDNA microarray in molecular subtyping of breast cancer [26].

In the present study, we found that luminal A subtype was the most prevalent followed by luminal B subtype, Triple Negative, and HER2-enriched derived exclusively. Our findings, from TMA IHC, are in contrast to most other studies performed in standard paraffin sections, in tissues from Sub-Saharan Africa particularly in west and central African countries where triple negative phenotype was reported to be the most common molecular subtype (43–82%) of breast cancer [16]. In Europe, North America, Asia and middle eastern countries, 30–70% of breast cancers are luminal A tumors and our result (40%) is comparable to the distribution seen in the western societies [27, 28]. However, Luminal B which is the second most common molecular subtype in our study (26%) is present at higher proportion among our study participants when compared to the rate seen in the Western high resource regions, Asia and middle east (10–20%) [27, 28].

A previous study among Ethiopian women carried out by Kantelhardt [20] in 2014 compared ER negativity in 352 patients out of 1208 consecutive patients treated at Addis Ababa-University Hospital, Ethiopia, from June 2005 through December 2010. They reported ER negative tumors to be around 35% in agreement with our present results [20]. A comparable result was also reported in a recent study done by groups at the University of Michigan in 2016 from one of the referral hospitals in Addis Ababa where they found ER negative tumors to be 26.5% [22].

A study among women of East African origin in US, in which a majority of the subjects (74%) were Ethiopians living in the United States, reported estrogen receptor negative tumors at 22% [29]. Similarly, recent studies in Kenya reported estrogen receptor negative tumor at 27.2% [21]. These few studies among East Africans, as well as our present study, which are summarized in Table 6 suggest the existence of geographical diversity in the distribution of molecular subtypes of breast cancer in Sub-Saharan Africa. These findings indicate that the frequency of ER negative tumors in East Africa may not be different from that found in the Western countries which is between 20 to 30% [15].

**Table 6:** Comparison of distribution of Molecular subtypes of Breast Cancer from Selected East African Studies

Author, year	Country	Number of breast cancer patients	Average age at diagnosis (yrs)	% ER−	% PR−	% HER2−	% ER− /PR− /HER2− (triple negative)
This Study, Endale et al.	Ethiopia	114	43	35	56	76	23
Jiagge et al., 2016 [22]	Ethiopia	94	43	–	–	–	15
Sayed S et al.,2014 [21]	Kenya	301	47.5	27.2	35.2	82.4^a^	20.2
Kantelhardt et al., 2014 [20]	Ethiopia	352	–	35	49	–	–
Jemal and Fedewa, 2012 [29]	USA^b^	186	48	22	35	–	–

^a^HER2 equivocal cases are considered as negative

^b^Done on Eastern Africa-born blacks living in USA (74% were Ethiopians)

Similarly the frequencies of triple negative tumors in these East African studies were lower than reported from west and central Africa. TNBC in the Kenyan study was reported in 20.2% of all cases [21] and TNBC in the Michigan study among Ethiopian women was reported to be around 15% which is lower than our finding (23%), but all of these studies indicate TNBC tumors to be less common than reported elsewhere in Africa. Hence, taken together, the data from East Africa suggest that it would not be accurate to associate African ancestry with increased probability of diagnosis with ER negative or TNBC tumors, as the Sub-Saharan African populations are known to be themselves highly heterogeneous in lifestyles, exposures, and genetic admixtures.

Present study has shown HER2 positivity at a slightly lower proportion than the rate seen among Ethiopians in the Michigan study [22] study (33%), the only other study (to our knowledge) which has incorporated HER2 assessment for molecular phenotyping of breast cancer from Ethiopia. However, HER2 positivity in our study (23%) is comparable to white Americans, African Americans, and West Africans which is about 17%, 19% and 20% respectively [22].

Our result shows an earlier onset of luminal B compared to the other molecular subtypes with statistically significant difference in median age at diagnosis (*P* < 0.05). The median age at diagnosis in our study also reveals a decline in luminal B subtypes as patients’ age increase with the incidence of luminal A exceeding that of luminal B at age ranges > = 50. This finding is comparable to a studies in the west where a bimodal age distribution at diagnosis is seen where incidence of the more aggressive phenotype luminal B peaks at earlier ages whereas luminal A type peaks at older ages [30].

The median age at diagnosis in our study was comparable to most previous studies in Africa and the few studies done among breast cancer patients in Ethiopia [20, 22]. However, it is much lower than the median age at diagnosis in most Western countries which is (55–60 years) [31]. The difference could be explained by the fact that African nations have younger population pyramids and the proportions reported in this and earlier studies are not age adjusted so the distributions are expected to always be shifted to younger ages in African cases compared to the cases in western countries; only 5% of African population is older than 60 years as compared to 24% of the population of Europe [32] being > 60 years old. More extensive population based registries in Africa, with active registration of cases, are urgently needed to understand the burden of cancer and to aid in cancer control program. Gaining a better understanding of environmental and lifestyle risk factors is crucial, as the incidence of breast cancer appears to be increasing at all ages in all African regions.

There was no statistically significant difference between median age at diagnosis and the ER status of breast cancer in this study. In this study, invasive ductal carcinoma was the predominant histological type (60%), which is comparable to the study by Kantelhardt in 2014 [20]. No statistically significant correlation was found between the molecular subtypes of breast cancer and histological type of breast cancer in this study, although the numbers of non ductal histologies were very low in the study. High tumor grade (Grade III) was reported in 34% of our study participants which is comparable with the study done by Kantelhardt [20]. The increased rate of high grade tumors observed in this study is in part possibly due to late diagnosis of breast cancer. No statistically significant correlation was found between the molecular subtypes of breast cancer and tumor grade in this study. The numbers of cases in each category were likely too small to appreciate differences, if they exist.

Limitations of the present study include selection bias which is due to enrollment of participants with available FFPE in the pathology laboratory, missing clinicopathological information and small sample size. Larger studies from population based samples are necessary to help guide cancer control programs. In spite of its stated limitations, our study adds strength to the notion that investments in utilization of hormonal and anti-HER2 therapies have potential to impact survival in Ethiopia, so efforts to spread education about implementing these strategies are recommended in light of the relatively high proportion of tumors amenable to these treatments.

## Conclusion

In conclusion, our study confirms the findings of studies from Ethiopia and other East African countries that hormone receptor negative tumors are not the most common molecular subtypes of breast cancer in this particular part of Africa. Hence, majority of breast cancer cases in this population may benefit from hormone therapy and/or anti-HER2 or other targeted therapy. Additionally, our findings and other East African Studies confirm the geographic variability in the distribution of the molecular subtypes of breast cancer in Africa and hence have important clinical and policy implications for breast cancer control. Furthermore, Ethiopian breast cancer patients exhibit highly proliferative Luminal B tumors at young ages. Future research should examine currently recognized as well as novel genetic and environmental factors that may contribute to the tumor characteristic differences between different populations in Africa.

## Abbreviations

ASCO/CAP: American Society of Clinical Oncology/College of American Pathologists
ER: Estrogen Receptor
FFPE: Formalin Fixed Paraffin Embedded
FISH: Fluorescence in-situ hybridization
HER2: Human epidermal growth factor receptor 2
IHC: Immunohistochemistry
Ki-67: Kiel 67
PR: Progesterone receptor
TNBC: Triple-negative breast cancer
